# Phylogenomic *Rhizobium* Species Are Structured by a Continuum of Diversity and Genomic Clusters

**DOI:** 10.3389/fmicb.2019.00910

**Published:** 2019-04-30

**Authors:** Víctor González, Rosa Isela Santamaría, Patricia Bustos, Olga María Pérez-Carrascal, Pablo Vinuesa, Soledad Juárez, Irma Martínez-Flores, Miguel Ángel Cevallos, Susana Brom, Esperanza Martínez-Romero, David Romero

**Affiliations:** Centro de Ciencias Genómicas, Universidad Nacional Autónoma de México, Cuernavaca, Mexico

**Keywords:** ecotype, evolution, genomic clusters, phylogenomics, plasmids, species, symbiosis, *Rhizobium*

## Abstract

The bacterial genus *Rhizobium* comprises diverse symbiotic nitrogen-fixing species associated with the roots of plants in the Leguminosae family. Multiple genomic clusters defined by whole genome comparisons occur within *Rhizobium*, but their equivalence to species is controversial. In this study we investigated such genomic clusters to ascertain their significance in a species phylogeny context. Phylogenomic inferences based on complete sets of ribosomal proteins and stringent core genome markers revealed the main lineages of *Rhizobium*. The clades corresponding to *R. etli* and *R. leguminosarum* species show several genomic clusters with average genomic nucleotide identities (ANI > 95%), and a continuum of divergent strains, respectively. They were found to be inversely correlated with the genetic distance estimated from concatenated ribosomal proteins. We uncovered evidence of a *Rhizobium* pangenome that was greatly expanded, both in its chromosomes and plasmids. Despite the variability of extra-chromosomal elements, our genomic comparisons revealed only a few chromid and plasmid families. The presence/absence profile of genes in the complete *Rhizobium* genomes agreed with the phylogenomic pattern of species divergence. Symbiotic genes were distributed according to the principal phylogenomic *Rhizobium* clades but did not resolve genome clusters within the clades. We distinguished some types of symbiotic plasmids within *Rhizobium* that displayed different rates of synonymous nucleotide substitutions in comparison to chromosomal genes. Symbiotic plasmids may have been repeatedly transferred horizontally between strains and species, in the process displacing and substituting pre-existing symbiotic plasmids. In summary, the results indicate that *Rhizobium* genomic clusters, as defined by whole genomic identities, might be part of a continuous process of evolutionary divergence that includes the core and the extrachromosomal elements leading to species formation.

## Introduction

Defining bacterial species remains a significant and controversial issue in biology (Woese, 1998; Cohan, 2001, 2002; Fraser et al., 2009; Shapiro and Polz, 2014; Bobay and Ochman, 2017). To correctly order the diversity of the bacterial world, and to distinguish the particular properties of pathogens, symbionts, and environmental bacterial isolates, it is crucial that a straightforward species concept is applied (Doolittle and Papke, 2006; Thompson et al., 2015; Bobay and Ochman, 2017). In practice, however, this is a challenging task, mainly due to the extraordinary variability of bacterial species and insufficient knowledge about their speciation mechanisms. In recent years, genomic technology has revealed bacterial species to be much more diverse than previously appreciated, with the added advantage that the genomic information is quantitatively comparable among isolates (Thompson et al., 2015). Moreover, by combining genomics with ecology and population genetics approaches, the taxonomy of several bacterial species has considerably advanced in several ways. For example: (i) Delineation of species and their boundaries within specific genera (Vinuesa et al., 2005; Pérez Carrascal et al., 2016; Ochoa-Sánchez and Vinuesa, 2017); (ii) Microevolutionary processes leading to speciation (Shapiro et al., 2012); (iii) Impact of recombination and HGT on species diversification (Cadillo-Quiroz et al., 2012; Shapiro et al., 2012; Bobay and Ochman, 2017).

In this paper, we study the taxonomy and evolution of *Rhizobium*, the nitrogen-fixing symbiotic bacteria associated with the roots of wild and domesticated legume plants. Several features of *Rhizobium* make this genus a formidable and demanding challenge in trying to determine its taxonomy. Chiefly among these, are (i) the sampling bias of *Rhizobium* strains, which come mainly (but not exclusively) from legume root nodules (Segovia et al., 1991; Miranda-Sánchez et al., 2016; Pérez Carrascal et al., 2016); (ii) the genome is partitioned into chromosomes, chromids, and plasmids, thought to foster ecological adaptations in various species of this genus (Galibert et al., 2001; González et al., 2006; Young et al., 2006; Shamseldin et al., 2016); and, (iii) the fact that large mobile plasmids—known as symbiotic plasmids, or pSym—and symbiotic islands encode most of the essential genes for root nodulation and nitrogen fixation (Johnston et al., 1978; Sullivan and Ronson, 1998).

A recent review on the status of *Rhizobium* taxonomy refers to 69 species isolated from distinct legume hosts (Shamseldin et al., 2017). Most of these species were classified by polyphasic taxonomy, including 16S ribosomal RNA gene sequencing, multilocus sequence analysis (MLS), biochemical properties, as well as phenotypic features. Currently, an alternative approach has been proposed, one based on nodulation host range and genomic average nucleotide identity (ANI) differences concerning to reference strains (Ormeño-Orrillo et al., 2015). For instance, following these criteria, the newly isolated nodulating bacteria *R. aegyptiacum* (Shamseldin et al., 2016), *R. esperanzae* (Cordeiro et al., 2017; Helene et al., 2017), and *R. ecuadorense* (Ribeiro et al., 2015) have been proposed as species. Similarly, Tong et al. (2018) suggested that *Rhizobium* genomic clusters as defined by ANI = 96%, corresponding to species. The basis for the concept was developed by Konstantinidis, Goris, and Roselló-Mora in several works proving strong correlations between experimental DNA–DNA re-association values when making pairwise whole-genome comparisons (Konstantinidis and Tiedje, 2005; Konstantinidis et al., 2006; Goris et al., 2007). In this respect, Richter and Roselló-Mora propose the ANI = 96% threshold explicitly, to define a bacterial species (Richter and Rosselló-Mora, 2009). Moreover, within species, ANI values can range widely, leading to either splitting or fusion of species depending on the cut-off used (Fraser et al., 2009). In short, while ANI is indicative of genomic clusters, we still need phylogenomic, ecological and population genetic criteria to delineate biologically meaningful species (Vinuesa et al., 2018).

*Rhizobium* species consist of multiple genomic lineages (Acosta et al., 2011; Ribeiro et al., 2013; Santamaría et al., 2017). Low recombination rates among strains of *R. etli* from diverse parts of the world indicate that differentiated genomic lineages may comprise a given species, or that they are, in fact, multiple species (Acosta et al., 2011). Sympatric populations of common bean nodulating *Rhizobium* and *R. leguminosarum* that nodulates clover, both consist of different genomic clusters of related strains, with low levels inter-cluster recombination (Kumar et al., 2015; Pérez Carrascal et al., 2016). Earlier work reported the coexistence of distinct *Rhizobium* species at the same site. In agricultural plots in France, Laguerre et al. (1993) described a *Rhizobium* population composed of three genomic species, one corresponding to *R. leguminosarum*, and two new species related to *R. etli* and *R. tropici*. In México, a sympatric pattern of several *Rhizobium* species also occurs, formed by the community assemblages of *R. etli–R. gallicum–Rhizobium* spp. (Silva et al., 2003) and *R. etli–R. phaseoli–Rhizobium* spp. (Pérez Carrascal et al., 2016; Miranda-Sánchez et al., 2016). Therefore, according to other genomic comparisons, those common bean-nodulating bacteria reported with strains assigned to *R. etli* or *R. phaseoli* may well correspond to independent species within the same agricultural plot (López-Guerrero et al., 2012; Miranda-Sánchez et al., 2016; Santamaría et al., 2017).

Despite many effort to define *Rhizobium* species with polyphasic taxonomy and genomic and phylogenomic approaches, the question concerning the biological meaning of genomic clusters within species and whether or not they are equivalent to species remains (Tong et al., 2018; Wang et al., 2018). We address this outstanding question, by carrying out a detailed genomic and phylogenomic comparison of recently released genomes of common-bean nodulating *Rhizobium* and a selected set of *Rhizobium* reference genomes available in GenBank. Our goal was to search for evidence of divergence that might be explained by evolutionary forces acting on core and accessory genes in the genomes examined.

## Materials and Methods

### Genome Collections

The initial compilation of 179 *Rhizobium* genomes was obtained from GenBank by 01-09-2016. Selected genomes were added to this compilation by inspection of the list of *Rhizobium* genomes in GenBank up to 20-11-2017. A total of 274 genomes were considered, of which 54 were complete, and 224 were drafts at distinct stages of completion. The final collection required to fulfill three criteria: (1) Genomes of *Rhizobium* strains that had been isolated exclusively from root nodules of wild and crop legume plants; (2) Complete or draft genomes had to contain the *nodABC* and *nifHDK* genes; (3) Draft genomes each had to have a total length >6 Mb. The length distribution of the 274 *Rhizobium* genomes indicated that those of 6–7 Mb were the most common. According to these criteria, a total of 87 *Rhizobium* genomes were obtained, including 44 complete genomes and 43 drafts. An additional complete genome of *Rhizobium* spp NXC12 (Miranda-Sánchez et al., 2016) that was generated in the course of this work was included in the final analysis, giving a total of 88 *Rhizobium* strains used. The NXC12 strain’s genome was determined by adopting the sequencing and assembly methodology already reported elsewhere (Bustos et al., 2017; Santamaría et al., 2017).

By conventional criteria, according to NCBI classification, this collection of 88 *Rhizobium* genomes belonged to 16 taxonomic species: *R. leguminosarum* (29 strains), *R. phaseoli* (15 strains), *R*. *etli* (7 strains), *R. gallicum* (3 strains), *R. leucaenae* (2 strains), *R. mesoamericanum* (2 strains), and single strains of *R. tropici*, *R. acidisoli*, *R. aegyptiacum*, *R. anhuiense*, *R. mongolense*, *R. ecuadorense*, *R. freirei*, *R. grahamii*, *R. sullae*, and *R. lusitanum*. Our collection also included 20 unclassified *Rhizobium* spp. strains. Additionally, for comparative purposes, the analysis considered eight genomes of *Sinorhizobium meliloti* strains, and likewise six of *S. fredii*. Supplementary Table S1 describes the main genomic features of the *Rhizobium* collection accompanied by their GenBank accession numbers.

### Phylogeny of Ribosomal, NodC, and RepC Proteins

The ribosomal, NodC, and RepC proteins were recovered from the *Rhizobium* and *Sinorhizobium* genome collection via BLASTp, by using a minimal similarity of 60%, minimal coverage of 60% of the largest protein, and an *e*-value threshold < 10^-6^. The 58 ribosomal proteins were concatenated and subject to multiple alignments with MUSCLE (Edgar, 2004a,b), and gaps removed with TrimAl v2.1 (Capella-Gutiérrez and Gabaldón, 2013). Phylogenetic trees were constructed by the maximum likelihood (ML) method based on the substitution matrix of JTT (Jones, Taylor, Thornton ref, date). Statistical significance of the tree was evaluated using 1000 bootstrap replicates, in MEGA-6 evolutionary analysis software (Tamura et al., 2013). To draw and edit the phylogenetic trees, we used FigTree software v1.4.3^1^. The same protocol was followed to build the phylogenetic trees of NodC and RepC protein families.

### Core Genome Phylogeny

A consensus core-genome was computed from 88 input *Rhizobium* genomes using the intersection of clusters computed by the bidirectional best-hit and Markov clustering (MCL) algorithms implemented in GET_HOMOLOGS (Contreras-Moreira and Vinuesa, 2013). The resulting 1069 core-genome clusters were processed with the GET_PHYLOMARKERS software package run in default mode (Vinuesa et al., 2018) to select those lacking evidence of recombination and horizontal gene transfer, excluding trees with anomalous or poorly resolved topologies. The codon alignments of loci passing these filters were concatenated to estimate an ML phylogeny under the best-fitting substitution model using IQ-TREE v1.6.1 (Nguyen et al., 2015).

### Genomic ANI Clusters Identification and Correlation Analysis

The Average Nucleotide Identity by MUMmer (ANIm) and the Genomic Coverage (G_cov_) values were calculated with the JSspecies program, with MUMmer used as a pairwise comparison tool for the collection of 102 genomes (Richter et al., 2016). The genetic distance parameter was obtained from the distance matrix underpinning the ribosomal phylogeny (see above). Briefly, estimates of evolutionary divergence between sequences (pairwise distances), were calculated according to the number of amino acid substitutions per site from between sequences. Analyses were conducted using the JTT matrix-based model. The analysis involved 102 amino acid sequences from 58 ribosomal proteins from each one of the 102 genomes. All positions containing gaps and missing data were eliminated. A total of 9727 positions remained in the final dataset. Evolutionary analyses were conducted in MEGA6.

The values of ANIm, Gcov, and genetic distance were used in Spearman (non-parametric) correlation tests. The Spearman *ρ* (rho) coefficient was calculated for sets of genomes belonging to the entire genome collection (i.e., 102 strains), for the *Rhizobium* genomes (88 strains), exclusively for clade C-I (*R. etli*, with 35 strains), and also clade C-II (*R. leguminosarum*, 38 strains). The Spearman test procedure was run in R with the package ggpubr (cor.test “spearman” and ggscatter for graphs) and adjusted to local regression^2^.

### Plasmid Comparative Genomics

MUMmer was used to implement plasmid pairwise comparisons, for which all the alignments > 300 bp were registered (Kurtz et al., 2004). Drawings for these were done with GeneVision, by taking the alignments obtained from MUMmer.

### Pangenome Modeling and Protein Clustering

To build the pangenome models we used the Bacterial Pangenome Analysis (BPGA) software package and its default values; that is, clustering via USEARCH with a minimal identity of 50% and 20 combinations (Chaudhari et al., 2016). Symbiotic genes included in the nodulation mutant database, or NodMutDB (Mao et al., 2005; Tian et al., 2012), were complemented by searching for symbiotic genes previously evaluated to obtain a total of 625 symbiotic genes. These known symbiotic genes were clustered with the Markov Clustering Algorithm (MCL), yielding 498 non-redundant symbiotic gene families. The symbiotic genes were mapped to the core and accessory pangenome components by BlastP Bi-directional Best Hits (BDBHs). The following BLASTp parameters were used to identify homologous proteins in the BDBHs: a minimum value of 50% for the amino acid identity, a minimum 50%-length in the alignment, and an *e*-value < 10^-6^. A presence/absence heat-map profile of symbiotic proteins in the 55 *Rhizobium* and *Sinorhizobium* complete genomes was drawn with the heatmap.2 function of the R’s gplots package.

### Rates of Synonymous Substitutions

We used the rate of neutral substitutions at the third codon position as a measure of the rate of divergence between pairs of genes. To do this, we first identified homologous proteins, by using the same BDBH criteria described above, in the chromosomes and symbiotic plasmids of selected *Rhizobium* genome pairs shown in Figure 7. These homologous proteins were retro-translated into the corresponding codons in the nucleotide sequence; then, the rate of synonymous substitutions at the third codon position was determined with the Ka/Ks calculator (Zhang et al., 2013).

## Results

### *Rhizobium* Phylogenomic Lineages

To investigate the major phylogenetic lineages and the species phylogeny of *Rhizobium*, we constructed a phylogenomic ML tree with 58 ribosomal proteins present in the genomes of 88 *Rhizobium* and 14 *Sinorhizobium* strains (Supplementary Table S1 and Supplementary Figure S1; see section “Materials and Methods”). The largest branches in the ribosomal tree connected four principal *Rhizobium* clades (rC-I to rC-IV) and two most distant clades that correspond to strains of *S. fredii* (rC-V) and *S. meliloti* (rC-VI), respectively. Likewise, the clades rC-I and rC-II contained predominantly strains of *R. etli* and *R*. *leguminosarum*, two early recognized species composed by multiple lineages. Moreover, the ribosomal clades rC-III and rC-IV harbor a low number of strains (9 and 6 strains, respectively) that represent very diverse *Rhizobium* species linked by large branches in the three.

**FIGURE 1:**
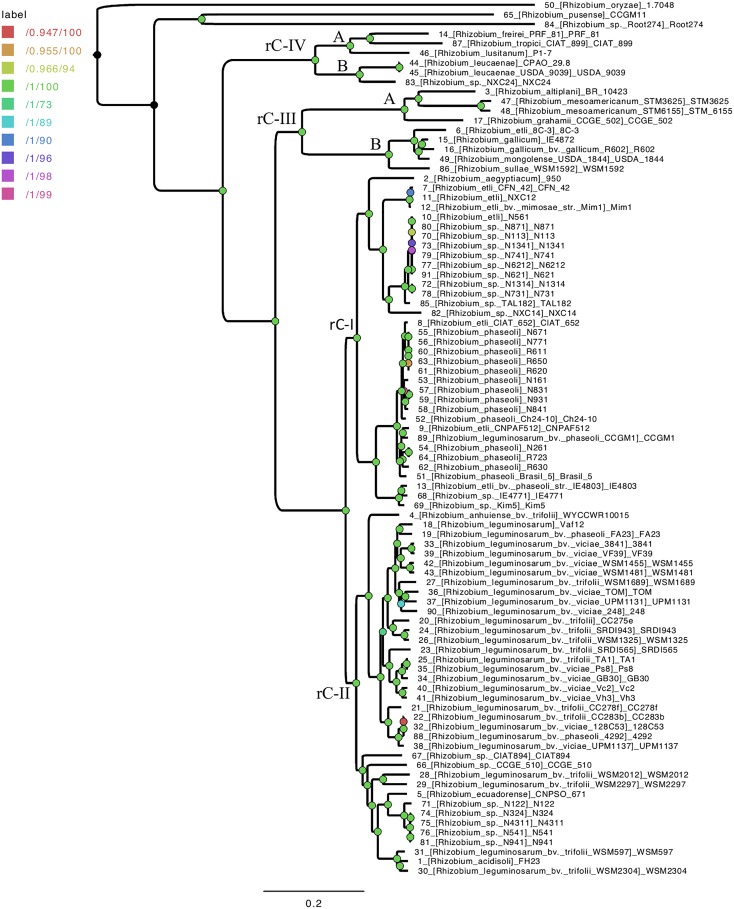
Species core genome phylogeny of 91 *Rhizobium* strains. Three additional *Rhizobium* strains were used here as outgroup for rooting: *R. oryzae* 1.7048, *R. pusense* CCGM11, and *Rhizobium* sp Root 274. The ML tree is based on consensus core proteins defined by the GET-HOMOLOGS package and selected for optimal phylogenetic attributes and the GET-PHYLOMARKERS tool. The nodes identified in the ribosomal tree (Supplementary Figure S1) that define the clades rC-I to rC-IV are labeled on the tree. The length and depth of the branches from the nodes indicated for clades rC-III and rC-IV, support two underlying clades (A and B) formed by highly divergent *Rhizobium* species.

Next, we inferred a species tree exclusively for the 88 *Rhizobium* genomes by using a stringent phylogenomic method to select core genes with optimal phylogenetic attributes (Vinuesa et al., 2018). The GET-HOMOLOGS package first defined a set of 1069 consensus core-genome clusters. Then, the GET_PHYLOMARKERS pipeline selected 437 top-scoring markers which were concatenated into a supermatrix, used to estimate a core-genome ML phylogeny (Figure 1). In this tree, *R. oryzae*, *R. pusense*, and *Rhizobium* sp. Root 274 were the most basal lineages found. The core-genome tree resolved the two large lineages holding species *R. etli* and *R. phaseoli* (clade rC-I), and *R*. *leguminosarum* (clade rC-II), also identified in the ribosomal tree. However, the phylogenomic core tree supports that the ribosomal clades rC-III and rC-IV are best defined as two independent and distantly related clades, each holding two major subclades: C-III-(A) containing *R. gallicum, R. mongolense*, and *R. sullae;* C-III-(B) formed with *R. mesoamericanum*, *R. altiplani*, and *R. grahamii*; C-IV-(A) for *R. leucaenae*, and C-IV-B for *R. tropici*, *R. freirei*, and *R. lusitanum*.

### Genomic ANIm Clusters Within Ribosomal Phylogenomic Clades

**FIGURE 2:**
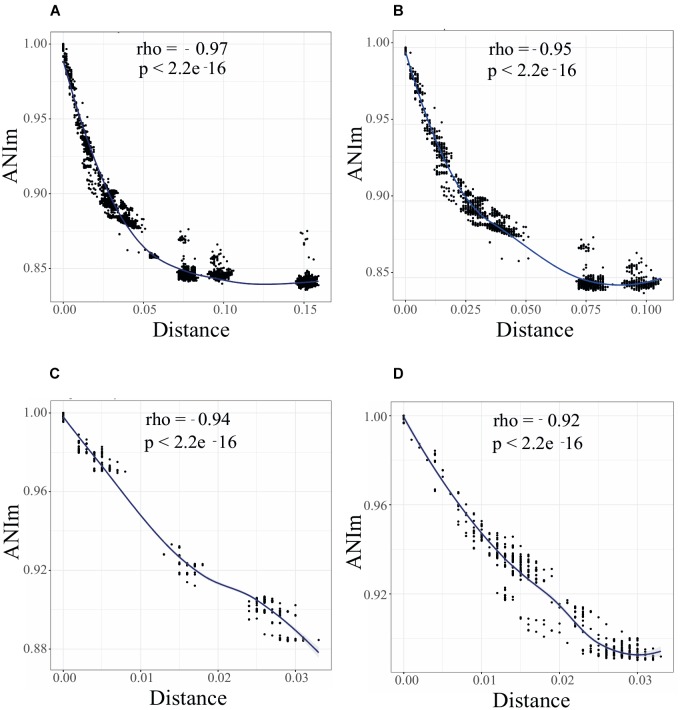
A continuum of genomic diversity and genome clusters in *Rhizobium* and *Sinorhizobium*. Spearman correlation test between ANIm and genetic distance between genome pairs of *Rhizobium* and *Sinorhizobium* strains: **(A)** 102 *Rhizobium* and *Sinorhizobium* strains, phylogenomic clades rC-I to rC-VI; **(B)** 88 *Rhizobium* strains of clades rC-I to rC-IV; **(C)** 35 strains of clade rC-I; **(D)** 38 strains of clade rC-II. Black dots indicate the Spearman correlations between pair of strains; local regression line in blue. ρ (R or Rho) and *P*-values are indicated in the inset.

To uncover the genomic clusters within *Rhizobium* clades, whole genomic comparisons using ANIm calculations were performed (see section “Materials and Methods”). Comparisons of ANIm results with respect to ANIb were similar using the same set of genomes (Supplementary Figure S3; Spearman *R* = 0.98, *p*-value =< 2.2e^-16^). They reveal an ample range of nucleotide variation among the 102 genomes studied here (*Rhizobium* and *Sinorhizobium*), with few closely related genomic clusters defined by the following criteria: ANIm > 96%, G_cov_ > 90%; there were also many individual genomes separated by ANIm < 96% (Supplementary Figure S2). As expected, the ANIm and G_cov_ of pairs of strains were inversely correlated with the genetic distances obtained for the building of the ribosomal tree: low genetic distances were associated with high ANIm values, and vice-versa (Figure 2A; Spearman correlationρ = 0.97, *p* < 2.1e^-16^; Supplementary Figure S4). There is also high correlation between ANIm and G_cov_ indicating small effects of horizontal transfer in ANI measures (Supplementary Figure S5).

The Spearman correlation plot depicted in Figure 2 showed a gradient of strains with an apparent continuum of genomic diversity, spanning few almost identical strains—ANIm > 98%; genetic distance 0.01 nucleotide substitutions per nucleotide site (JTT neutral model)—to those strains with ANIm values up to ANIm 88% and genetic distance 0.05. Moreover, the Spearman correlation slope fell to an asymptote when the genetic distance increased at ranges higher than 0.075 showing no correlation with lower pairwise ANIm about 80–85%, probably due to saturation of nucleotide substitutions at a high genetic distance. These three well-separated groups included strains of the ribosomal clades rC-III to rC-VI, that are the most distantly related strains also found in the core phylogenomic tree (Supplementary Figure S1). When the Spearman test was applied to only to the genomes of the 88 *Rhizobium* strains, there is a straight continuous line of ANI vs. genetic distance correlation, and two uncorrelated groups at higher genetic distances (belonging to *Rhizobium* clades rC-III and rC-IV) (Figure 2B; Supplementary Figure S1). Therefore, the straight line of correlation contains many of the strains of the clades rC-I and rC-II, and few strains of rC-III and rC-IV that appear in the asymptote as two separated clusters of distantly related strains (Figure 2). More importantly, Figure 2C shows that within the clade rC-I three well-separated clusters represent the ANIm relationships between subclades (Supplementary Figures S1, S6). By contrast, a clear genomic cluster separation did not occur in the *R*. *leguminosarum* clade rC-II (Figure 2D).

The distribution of ANIm per segments of 2% identity showed several peaks of cumulative values (Supplementary Figure S6). The highest peak in the plots corresponds to comparisons between ribosomal clades; small peaks represent comparisons between genomes of the same ribosomal clade (Supplementary Figure S6). In the range of ANIm = 95–97% there were few pair-wise ANIm values when the six ribosomal clades (rC-I to rC-VI), or only four ribosomal clades were compared (rC-I to rC-IV); Supplementary Figures S6A,B). The genomes of strains of the ribosomal clade rC-I showed a cluster of discrete peaks at ANIm values > 97%, and absence of registered ANIm values within 94–97%. The peaks of ANI > 97% corresponded to the genomes of *Rhizobium* strains within subclades in the rC-I clade (Supplementary Figure S1, small circles inside rC-I). The ANIm relationships between the ribosomal subclades accumulated in three peaks around ANIm 90 and 92% (Supplementary Figure S6E). In contrast, the ANIm values of the genomes of the ribosomal clade rC-II were distributed along the scale of ANIm of 88–99% showing a continuum of diversity, with few pairs of genomes with ANIm superior to 96% (Supplementary Figures S6D,F).

### Mapping the Accessory Genome Within *Rhizobium* Phylogenomic Clades

**FIGURE 3:**
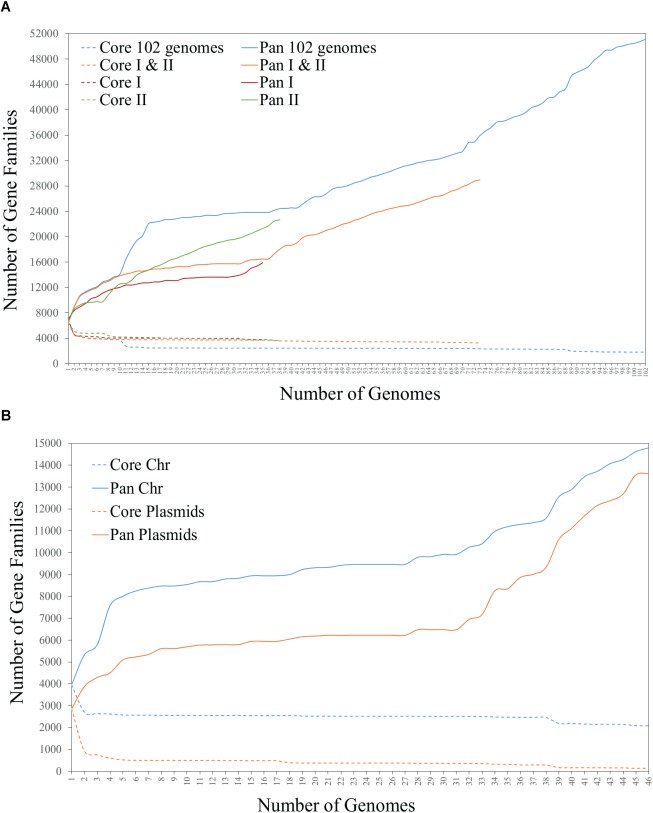
Core and pangenome BPGA models of **(A)** 102 genomes of *Rhizobium* and *Sinorhizobium*, and 73 genomes of the strains of *Rhizobium* clades rC-I and rC-II, and **(B)** chromosome and plasmids of the 45 complete *Rhizobium* genomes.

To determine if the variability in accessory genes corresponds to the phylogenetic divergence of *Rhizobium* clades, we built several pangenome models based on the 102 *Rhizobium* and *Sinorhizobium* (see section “Materials and Methods”). The BPGA models showed a very extended pangenome, with a small core and a considerably high accessory component. The pangenome profile remains the same when restricted to the 88 *Rhizobium* strains (not shown), the single clades rC-I and rC-II, or the latter two together (Figure 3A; core and pan I and II). Furthermore, when pangenome models were constructed only for chromosomes, or only for extrachromosomal elements from 45 *Rhizobium* complete genomes of the collection, they showed extreme variability in the extrachromosomal accessory content, with a minimal core of 64 protein-coding families (Figure 3B). This comparison also revealed that despite the chromosomal core remaining almost equal after adding new individual genomes, the chromosomal accessory component was similar in size to that of the extrachromosomal accessory component.

**FIGURE 4:**
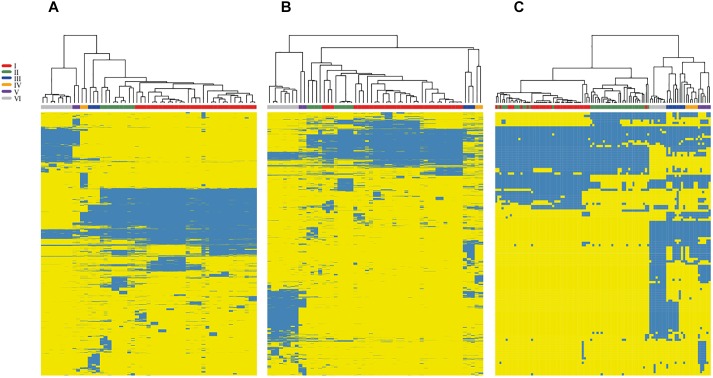
Variation profile in the accessory genome of *Rhizobium* by mapping onto chromosomes and plasmids. The heat-maps show the profile of accessory gene obtained separately for chromosomes **(A)** and plasmids **(B)** of 55 complete genomes of strains and species of *Rhizobium*. Blue indicates the presence and yellows the absence of the corresponding gene. A symbiotic gene profile **(C)** was obtained by checking the BDBHs of 201 symbiotic genes registered in the NodMutdb curated database against the complete set 102 genomes of *Rhizobium* and *Sinorhizobium*. Labels in colors indicate the ribosomal clades of the trees of Figure 1 and Supplementary Figure S1.

Next, we mapped the distribution of accessory genes onto the chromosomes and plasmids in the selected set of 45 complete genomes of *Rhizobium* and 10 of *Sinorhizobium* (Figure 4). The heat-map profile for the presence/absence of chromosome-specific accessory genes only in the chromosomes, distinct strains with low Bray Curtis dissimilarity within the phylogenomic clades (rC-I to rC-VI) and, higher Bray Curtis between clades (Figure 4A and Supplementary Table S2). The accessory genes mapped exclusively onto the plasmids of 55 complete genomes look similar to the chromosomal profile. However, three strains (MIM1, NCX12, and NXC14) considered within the clade rC-I, are inserted within the clade rC-II. These strains of the clade rC-II contain accessory genes seemingly present in the clade rC-I (Figure 4B). Such discrepancy may be due to events of HGT between strains.

To determine if the symbiotic genes also agree with the main phylogenomic ribosomal clades, we analyzed the distribution of 221 symbiotic genes that exclusively match by BDBHs with the 102 genomes of *Rhizobium* and *Sinorhizobium* (see section “Materials and Methods”). Overall the profile is comparable to the heat-maps of accessory genes in chromosomes and plasmids (Figure 4C and Supplementary Table S2). Markedly, there are several inconsistencies between the Bray-Curtis dissimilarity group that comprises the clade rC-I. Within the clade rC-I there are insertions of some strains that belong to the clade rC-II. This pattern may be due to inter strain mobilization of the symbiotic plasmid genes or the complete plasmid frequently present in strains of the clade rC-I. This observation is consistent with ample evidence already published of the mobile nature of this symbiotic replicon (Pérez-Mendoza et al., 2004; Pérez Carrascal et al., 2016).

### Limited Number of *Rhizobium* Extrachromosomal Families

**FIGURE 5:**
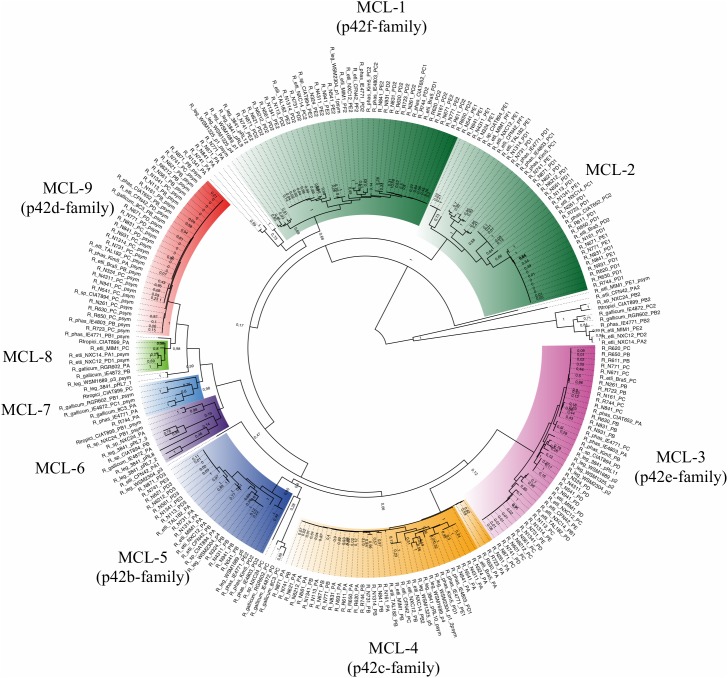
Phylogenetic families of RepC. The RepC proteins from complete *Rhizobium* genomes were identified by BLASTp and clustered by MCL to define the homologous groups. Phylogenies were constructed with the maximum likelihood method using the JTT matrix, and a bootstrap of *n* = 1000 replicates (see section “Materials and Methods”). Colors indicate the phylogenetic clades and their correspondence with the RepC-MCL clusters. They are also indicated by the text around the circle with the corresponding plasmid family.

**FIGURE 6:**
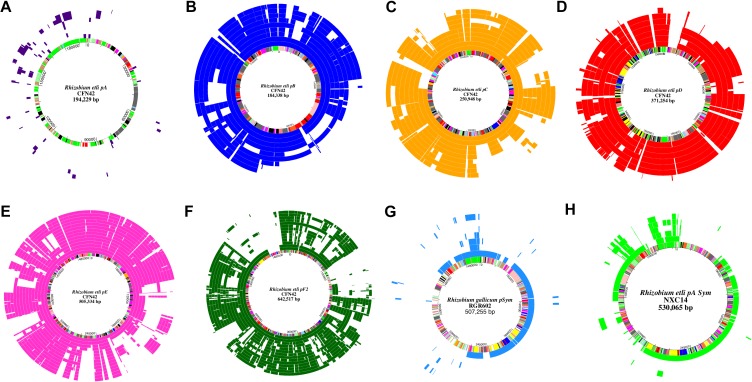
Conserved families of chromids and plasmids in *Rhizobium*. Pairs of homologous plasmids were identified by their closest phylogenetic relationship in the RepC tree. Then, MUMmer matches longer than 300 bp were aligned with respect to the plasmids of *R. etli* CFN42 **(A–F)**, *R*. *gallicum* R602 **(G)**, and *Rhizobium* sp NXC14 **(H)**, located in the innermost circle with color bars indicating gene functional annotation according to González et al. (2006). Figure was drawn with Genevision. Order of the elements in the schemes from the innermost to the outermost circle are the next. **(A)**. CFN42pA. Purple: R.leg.3841pRL7, LPU83pII, IE4872pA, CIAT894pB, R.leg.3841pRL8, NXC24pB, CIAT899pB. **(B)** CFN42pB. Blue: NXC12pA, Mim1pA, IE4803pD, Kim5pD, Tal182pA, IE4771pE, WSM2304p4, CIAT894pA, WSM1689p5GR4pB. **(C)** CFN42pC. Gold: NXC12pB, Mim1pB, IE4803pD, IE4771pE, Kim5pD, 3841pRL10, WSM1325p5, WSM2304pRLG201, WSM1689p4. **(D)** CFN42pD. Red: CIAT652pB, 8C3pB, Tal182pC, Kim5pA, CIAT894pC, Bra5pB, IE4771pB. **(E)** CFN42pE. Pink: WSM1689p2, NXC12pC, Mim1pD, Tal182pD, NXC14pB, CIAT894pD, CIAT652pA, Kim5pB, IE4771pC, Bra5pC, IE4803pA, WSM2304pRLG202, WSM1325p2, 3841pRL11. **(F)** CFN42pF2. Green: NXC12pE, Mim1pF, IE4771pD, WSM1689p1, WSM1325p1sym, R.leg.3841pRL12, WSM2304pSym, CIAT652pC, CIAT894pE, Tal182pE, Kim5pC, IE4803pC, Bra5pD, NXC14pC, WSM1325p4, 3841pRL9. **(G)** R602 pSym. Light blue: IE4872pSym, IE4771pA, 8C3pB, R744pA, CIAT899pC, LPU83pV, Rm41pA. **(H)** NXC14pA pSym. Light green: NXC12pSym, CIAT899pA, IE4872pB, Mim1pC, R602pA, CFN42pF1, NXC12pE, Mim1pF, CIAT652pC, IE4771pD, CIAT894pE, Tal182pE, Kim5pC, IE4803pC, Bra5pD.

Despite the variability found in extrachromosomal elements, the number of chromid or plasmid families remains unknown. It has been suggested that the plasmid replication protein (RepC) and the plasmid partition proteins (RepA, RepB) might reflect the diversity of chromids and plasmids (Cevallos et al., 2008). Based on this assumption, we identified 514 conserved protein sequences of RepC, and 508 of the RepB in 102 *Rhizobium* and *Sinorhizobium* genomes. Phylogenetic ML trees produce very similar results using any of these proteins (Figure 5 and Supplementary Figure S7). Secondly, all the RepC proteins were clustered with MCL into nine large clusters (see section “Materials and Methods”). The tree in Figure 5 contains several divergent clades of closely related RepC proteins that also correspond to MCL clusters, suggesting the possibility that they correspond to conserved plasmid families (Figure 5). The same pattern is observed for the RepB phylogenetic tree despite of lower diversity of this protein (Supplementary Figure S7). Six MCL clusters consist of closely related phylogenetic clades and included the RepC of the chromids p42e and f, and the plasmids p4a to d (symbiotic plasmid) of *R. etli* CFN42 (MCL-clusters 1, 3-5, and 9). Other 3 MCL clusters (MCL-6 to 8) were assemblies of different and divergent RepC weakly related to each other. MCL-2 contains homologs to the RepC-2 of the plasmid p42f that also contains the replicator RepC-1 (González et al., 2006). To assess the whole DNA conservation of the conserved RepC clades, the complete DNA sequences of some plasmids within the clades were compared by MUMmer with the chromids and plasmids p42b to p42f of *R. etli* CFN42 (Figure 6). The comparison showed five families concerning to *R. etli* CFN42 that are remarkably conserved in sequence (Figure 5, 6B–F). Other minor RepC clades included different RepC proteins from plasmids corresponding to species of *R. gallicum*, *R. tropici*, and *Sinorhizobium*. These latter RepC families were poorly conserved in the whole DNA sequence.

### Symbiotic Plasmids (pSyms) Within *Rhizobium*

**FIGURE 7:**
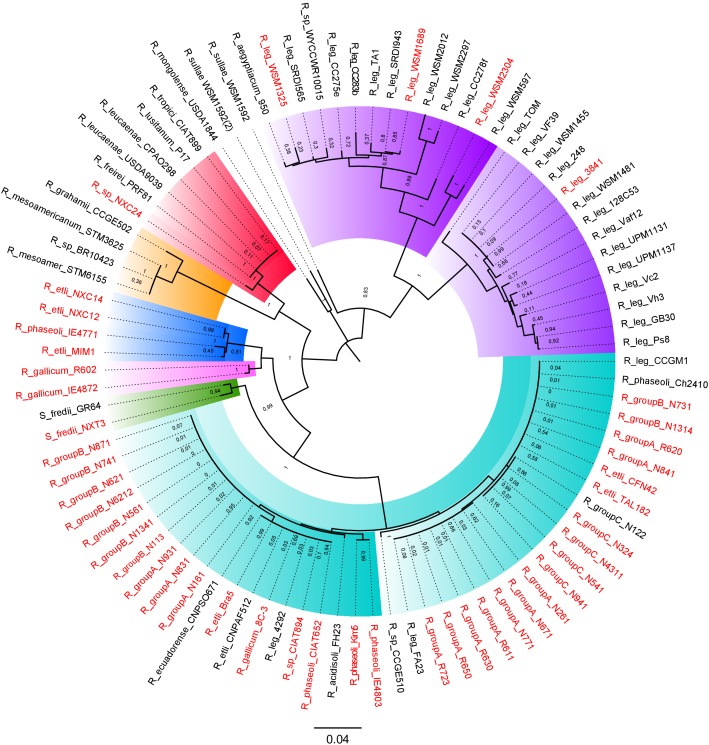
Phylogenetic tree of NodC from the *Rhizobium* plasmids. NodC proteins from 45 complete *Rhizobium* genomes were first identified by BLASTp. Phylogenies were estimated with the maximum likelihood method with the JTT matrix and a bootstrap of *n* = 1000 replicates (see section “Materials and Methods”). In red color, strains with completely sequenced pSyms are indicated.

To identify the symbiotic plasmids variants present in our collection, we inferred an ML phylogeny of the common nodulation NodC protein, a chitin-oligosaccharide synthase essential for the production of the nodulation factor common to all known symbiotic plasmids. The ML phylogeny resolved seven distinct major NodC clades in *Rhizobium* (Figure 7). The phylogeny, consistent with the taxonomic definition of *Rhizobium* species, contains a very well represented clade that belongs to NodC of the pSym of *R*. *etli* CFN42 characterized earlier. This pSym is mainly distributed in strains of rC-I, but also in some *Rhizobium* strains of clade rC-II and rC-IV, as *Rhizobium* sp. CIAT894 and *R. gallicum* IE4872. Despite its wide distribution, this pSym showed high sequence conservation (Figure 6D; González et al., 2003; Pérez Carrascal et al., 2016). Indeed, within the *Rhizobium* clade rC-I some strains harbored distinct types of pSym. For instance, the strain IE4771 isolated from the common bean, and MIM1 isolated from *Mimosa* nodules but with the capacity to nodulate common bean, had distinct types of pSyms. The pSym of MIM1 strain shares similar large regions with the pSym of *Rhizobium* NXC12 and NXC14 strains isolated from Huautla, México (Figure 6H).

The NodC phylogeny separates the symbiotic plasmids of *R. leguminosarum* (ribosomal clade rC-II) in two different clades. One of these contained plasmids of the well-studied *R. leguminosarum* 3841, TOM, and VF39 model strains that nodulate the Leguminosae genera *Vicia*, *Lathyrus*, *Lens*, and *Pisum*. The second clade included different pSyms found in strains isolated mainly from *Trifolium* recovered worldwide. Although the NodC of the pSyms of *R. leguminosarun* were grouped in a single very divergent clade, they do not represent a single structurally conserved plasmid (Figure 7). The plasmid pRL10 (the pSym of the strain 3841) showed high conservation in sequence with respect to p42c (non-symbiotic plasmid) of *R. etli* CFN42 (Figure 6C; Crossman et al., 2008). Figure 6F shows the comparison of the pSyms of the *R. leguminosarum* strains WSM1325 and WSM2304 respect to the p42f. Additional ANI comparisons performed only with the updated complete sequences of several pSyms of *R. leguminosarum* indicate low overall similarity between them (Supplementary Figure S8). Unidirectional BLASTp of the total predicted proteins of the pRL10 vs. all the other enoded in the complete *R*. *leguminosarum* pSyms indicate the conservation of the *nif-nod* region despite low conserved proteins in the rest of the pSyms (Supplementary Figure S9). Therefore, these instances support the hypothesis that either the *nif-nod* region might be transposable between replicons or it was acquired independently (Crossman et al., 2008). Other *Rhizobium* pSyms with some large conserved regions belong to *R. gallicum* (R602 and IE4872; Figure 6G), and *R. tropicii* (NXC24 and CIAT899). The results suggest that distinct mechanisms underlie the evolution of the pSyms within the *Rhizobium* clades.

### Rates of Evolution of Symbiotic Plasmids

**FIGURE 8:**
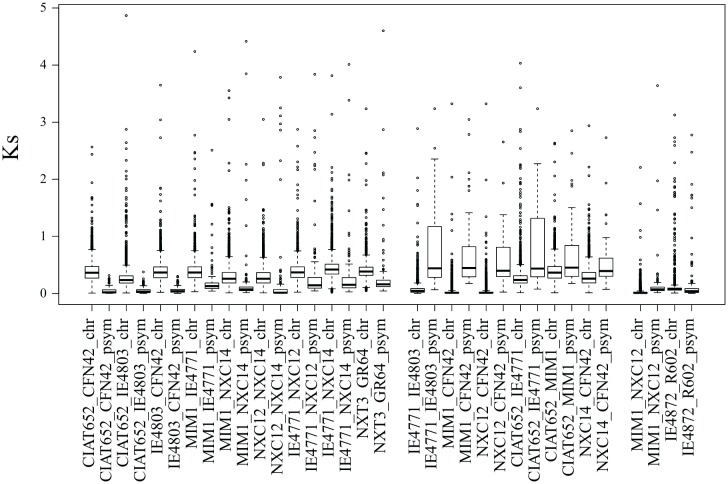
Relative rates of evolution of symbiotic plasmids in relation to chromosomes. Rates of synonymous substitutions (*Ks*) were estimated, for the common genes found from chromosomes and symbiotic plasmids, between pairs of *Rhizobium* strains. *Ks* was calculated with the Ka/Ks calculator (Zhang et al., 2013), an evolutionary analysis software tool.

Horizontal transfer of pSyms has been proposed to occur repeatedly in *Rhizobium* evolution (Martínez-Romero, 2009; Rogel et al., 2014; Zahran, 2017). In the process, owing to displacement and substitution effects, incongruent evolution rates between the chromosome and pSym are expected. To test this, we contrasted the rates of synonymous substitutions (*Ks*) in conserved genes of chromosomes and pSyms between pairs of *Rhizobium* strains. The third nucleotide position in the codons is free to change during short evolutionary periods because it is generally selectively neutral. Hence, *Ks* rates are a proxy for the rates of evolution since the divergence of gene pairs. For instance, the pairs of strains of *R. etli* and *R. phaseoli*, CFN42-CIAT652, NXC12-NXC14, Mim1-NXC14, and CIAT652-4803, have *Ks* values close to zero for the symbiotic plasmid, whereas chromosomal genes had *Ks* > 0.5 (Figure 8). Thus, symbiotic plasmids seem to be a recent addition to the genomes of these paired strains. More distant pairs of strains (e.g., CIAT652-IE4771, CIAT652-Mim1) had higher symbiotic plasmid *Ks* rates than did the chromosomal genes, indicating dissimilar evolutionary lines of these compartments since the paired strains diverged. Finally, we found two examples of low *Ks* rates for chromosome and symbiotic plasmids in the *R. gallicum* IE4872-R602 pair, and in the *Rhizobium* Mim1-NXC12, suggesting that both genome compartments have comparable evolutionary histories.

## Discussion

The concept of new biological species (speciation) dates back to Darwin, posing problems for many past and contemporary evolutionary biologists (Mallet, 1995, 2008; Shapiro et al., 2016). Recently, some authors have argued for the concept of speciation as a continuous process over time with several stages eventually leading to distinct phylogenetic lineages or species (Lowry, 2012; Seehausen et al., 2014; Lowry and Gould, 2016). In this study, we investigated the phylogenetic meaning of genomic ANIm clusters, through global genomic comparisons within known *Rhizobium* species, and whether they are equivalent to species or instead represent a point in the course of speciation. The significance and limits of genomic clusters for defining species were discussed before into the light of the correspondence between experimentally determined DNA-DNA hybridization (DDH) of 70% with 96% ANI values (Konstantinidis and Tiedje, 2005; Goris et al., 2007; Richter and Rosselló-Mora, 2009). In particular, Konstantinidis et al. (Konstantinidis and Tiedje, 2005) distinguished ecological milieu and niche adaptation as two factors influencing the formation of clusters of closely related strains. Obligatory pathogenic species, such as *Staphylococci* and *Streptococci*, with narrow ecological niches, are organized in well-differentiated clusters, whereas free-living bacteria from the ocean or soil typically lie on a continuum of diversity, with their genomic clusters organized at differing levels of similarity. Recently, Ochoa and Vinuesa (Ochoa-Sánchez and Vinuesa, 2017) defined ecologically and genetically coherent species in the *Stenotrophomonas* genus without any pre-determined genomic identity cut-off. Instead, they applied a combination of phylogenetic and population genetics approaches to delimit species. Despite these observations, the pragmatic definition of species relying on genomic ANI comparisons has dominated the genomic era (Ciufo et al., 2018). A recent study, powerfully supported by high-resolution ANI comparisons of more than 90,000 genomes, reinforce the ANI 95–96% as the delimiting value for operational definition of species (Jain et al., 2018).

We found several genomic clusters at different ranks of ANI and genetic distance within the main evolutionary lineages of the *Rhizobium* genus. There were groups of *Rhizobium* species (rC-III and rC-IV) underrepresented in our collection. They were both phylogenetically distant and appeared in the lower range of ANIm and G_cov_ (Figure 2 and Supplementary Figures S4, S5). At the other extreme of the ANI ranges, with ANIm of 96% as a cut-off, some genomic clusters were found. They agree with similar genomic clusters reported elsewhere proposed to represent new species (Tong et al., 2018). However, overall the *Rhizobium* strains within these clades show a continuum of ANIm values inversely correlated with genetic distance (Figure 2A,B). In the analysis presented here, the species *R. etli* and *R. leguminosarum* within the ribosomal clades rC-I and rC-II, respectively, comprises strains related at ranges of ANIm 90–98% (Figure 2C,D). In rC-I some genomic clusters were detected at ANI > 96%, but in rC-II there is not a clear ANIm cut-off to define species without the complementary phylogenetic analysis.

A recent study by Jain et al. (2018) challenges the model of a continuum of genetic diversity along the formation of species and supports the concept of species delimitation by ANI > 95% and interspecies below ANI 83%. The scale at which this experiment was done is not comparable to the study reported here. It is possible that at small scale comparisons the discontinuity between ANI > 83% < 95% observed by Jain et al., appear to be less pronounced due to species subsampling (Jain et al., 2018). In *Rhizobium* the mobile nature of the symbiotic plasmids might explain that *Rhizobium* populations contain wide range of chromosomal diversity but narrow symbiotic variation (Pérez Carrascal et al., 2016).

Since the accessory core component of the *Rhizobium* pangenome is thought to be the outcome of local adaptation to contend with particular ecological conditions, we expected to find marked genetic differences between genomic clusters if they represent ecologically differentiated units. The distribution of accessory genes (including the symbiotic genes) in the 55 complete genomes of *Rhizobium* and *Sinorhizobium* showed patterns of gene presence and absence consistent with the phylogenetic diversification of six major clades identified in the ribosomal phylogenomic tree (Supplementary Figure S1). Accordingly, we found that the number of RepC families of chromids and plasmids was small and less diverse than suggested by electrophoretic patterns of *Rhizobium* isolates (Figure 5) (Jumas-Bilak et al., 1998; Mazur et al., 2011; Zahran, 2017). The apparent high plasmid diversity in *Rhizobium* probably arose from DNA rearrangements, like replicon fusion or fissions, as well as from insertions and deletions (González et al., 2010; Pérez Carrascal et al., 2016). There is no doubt that such genetic processes occur in *Rhizobium* (Flores et al., 1988, 1993; Romero et al., 1991; González et al., 2010).

Understanding the rates of HGT and the evolution of pSyms is of practical importance for agricultural management (Sullivan and Ronson, 1998; Remigi et al., 2016). *Rhizobium* species contain different pSyms, as described here, which may become displaced and substituted by another compatible pSym (Martínez-Romero, 2009). Underpinning this concept are the two distinct types of symbiotic plasmids observed in the closely related pair of *R. etli* strains CFN42 and Mim1 (Ormeño-Orrillo et al., 2015). In our study, we found that IE4771, IE4803 (Silva et al., 2003), and the pair of *R gallicum* R602 and 8C3, misclassified in GenBank probably underwent a pSym displacement event (Bustos et al., 2017). Indeed, *R. aegyptiacum* within the *Rhizobium etli* clade rC-I harbors a pSym typically found in the *R. leguminosarum* strains nodulating *Trifolium* roots (Shamseldin et al., 2016). Horizontal transfer of pSyms to phylogenetically distant species produces discrepancies in neutral evolution rates between the chromosome and recently introduced pSyms, which would indicate an ongoing process of symbiotic transfer (Andrews et al., 2018). Our results highlight that the pSym, prevalent in *R. etli* and *R. phaseoli* (González et al., 2003), likely has a recent origin given its high conservation and low rates of divergence in synonymous codon positions, a conclusion also supported by a recently published analysis (Li et al., 2018).

Indeed, multiple species concepts could be applied based on evidence of genomic similarity shared ecological niches, or functional data such as recombination (Lowry and Gould, 2016; Bobay and Ochman, 2017; Cohan, 2017; Jain et al., 2018; Shapiro, 2018). A population genomics criterion for species definition adds to the similarity concept of species, the notion of a process driven by evolutionary, ecological, and molecular genetics mechanisms (Shapiro, 2018). Genomic clusters delimited by ANI > 95% are currently applied to define taxonomic species (Ciufo et al., 2018). However, we suggest that genomic clusters are part of an evolutionary continuum within the species defined by the phylogenomic clades. In other words, phylogenomic clades might be equivalent to species, and genomic clusters and the strains distributed at different ranges of ANIm represent stages or points along the species diversification. Strain diversification within clades likely originates genomic clusters isolated by association to particular ecological niches, recombination, horizontal gene transfer, or genomic identity. We believe that robust identification and association of genomic clusters undergoing the speciation process is a task best performed with population genomics approaches (Shapiro, 2018). Application of genetic tests for recombination, genetic differentiation, and gene flow, and the incorporation of multispecies coalescent models to well sampled bacterial populations could define the significant ANI value to delimit lineages and species (Ochoa-Sánchez and Vinuesa, 2017). However, the problem of species borders remains as one of the major issues in contemporary bacterial evolution and taxonomy.

## Author Contributions

VG conceived the study through discussion with the group formed by MC, EM-R, DR, PV, and SB and coordinated and analyzed the experimental and bioinformatics work and wrote the draft manuscript. RS and PB performed the phylogenetic, pangenome, comparative genomics, and statistical analyses. OP-C conducted the primary experiments on *Rhizobium* genome comparisons, and review the draft manuscript. IM-F compiled the required information from PubMed that extend the collection of symbiotic genes of the NodMutDB. SJ carried out experimental sequencing methods. MC, EM-R, DR, PV, and SB contributed bacterial strains and their sequencing results and reviewed the manuscript.

## Conflict of Interest Statement

The authors declare that the research was conducted in the absence of any commercial or financial relationships that could be construed as a potential conflict of interest.

## Footnotes

1 http://tree.bio.ed.ac.uk/software/figtree

2 https://CRAN.R-project.org/package=ggpubr

## Supplementary Material

FIGURE S1 Phylogenetic tree of ribosomal proteins of *Rhizobium* species. Ribosomal clades are indicated by Roman numerals (I to VI) enclosed in colored ellipses. Subclades appear with black ellipses within the clades. The tree was constructed using maximum likelihood in the MEGA software package, by using 58 concatenated ribosomal proteins (refer to section “Materials and Methods” for more details).

FIGURE S2 Genomic clusters of 102 *Rhizobium* and *Sinorhizobium* pairs obtained by pairwise ANIm comparisons.

FIGURE S3 Correlation between ANIm and ANIb in pairwise comparison of 102 *Rhizobium* and *Sinorhizobium* genomes. Spearman *r* and *p*-values are shown.

FIGURE S4 Correlation between G_cov_ and phylogenetic distance. **(a)** 102 *Rhizobium* and *Sinorhizobium* genomes, **(b)** 88 genomes, **(c)** 35 genomes of rC-I clade, and **(d)** 38 genomes o rC-II clade. Spearman *r* and *p*-values are shown.

FIGURE S5 Correlation between ANIm and G_cov_. **(a)** 102 *Rhizobium* and *Sinorhizobium* genomes, **(b)** 88 genomes, **(c)** 35 genomes of rC-I clade, and **(d)** 38 genomes o rC-II clade. Spearman *r* and *p*-values are indicated in the inset.

FIGURE S6 Distribution of ANI values between pairs of genomes belonging to **(a)** 102 genomes, **(b)** 88 genomes, **(c)** and **(e)** 35 genomes of rC-I clade, and **(d)** and **(f)** 38 genomes o rC-II clade. Histograms show the frequency of pairwise comparisons within clades (turquoise color), and inter clades (pale red), distributed by segments of ANI values. In figures **(e)** and **(f)**, blue color histograms show comparisons within subclades (see Supplementary Figure S1) defined by ANIm > 95%, and the green color histograms indicate inter subclades comparisons.

FIGURE S7 Phylogenetic families of RepB. Phylogenies were constructed with the maximum likelihood method and a bootstrap of *n* = 1000 replicates. Colors indicate the phylogenetic clades and their correspondence with the MCL clusters determined as indicated in the section “Materials and Methods” and Figure 5.

FIGURE S8 Pairwise ANIm comparison between complete *R. leguminosarum* symbiotic plasmids. Only the genome sequences of *R. leguminosarum* listed in the GenBank up to 14-03-2019 were downloaded. The ANIm comparison were performed using JSspecies as described in the methods section. The heatmap show the values of the corrected G_cov_% / ANIm%; the color scale key inset shows the ranges from 0 to 1.

FIGURE S9 Comparison of the complete proteomes of the symbiotic plasmids of *R. leguminosarum*. Unidirectional BLASTp using the total predicted proteins of *R. leguminosarum* 3841 pRL10 symbiotic plasmid (illustrated by the red innermost circle) against the predicted proteins of others symbiotic plasmid indicated in the inset. Blast hits were recorded and showed in black circles. The *nif-nod* region is indicated by the yellow bars in the red inner circle.

TABLE S1 The collection of genomes of *Rhizobium* and *Sinorhizobium* used in this study.

TABLE S2 Bray Curtis dissimilarity index for accessory genes distribution within and between ribosomal clusters.
