# Supplementary figures and images for: Phylogenomic Rhizobium Species Are Structured by a Continuum of Diversity and Genomic Clusters
Source: Front Microbiol. 2019 Apr 30;10:910. doi: 10.3389/fmicb.2019.00910 (PMC6503217; doi:10.3389/fmicb.2019.00910)

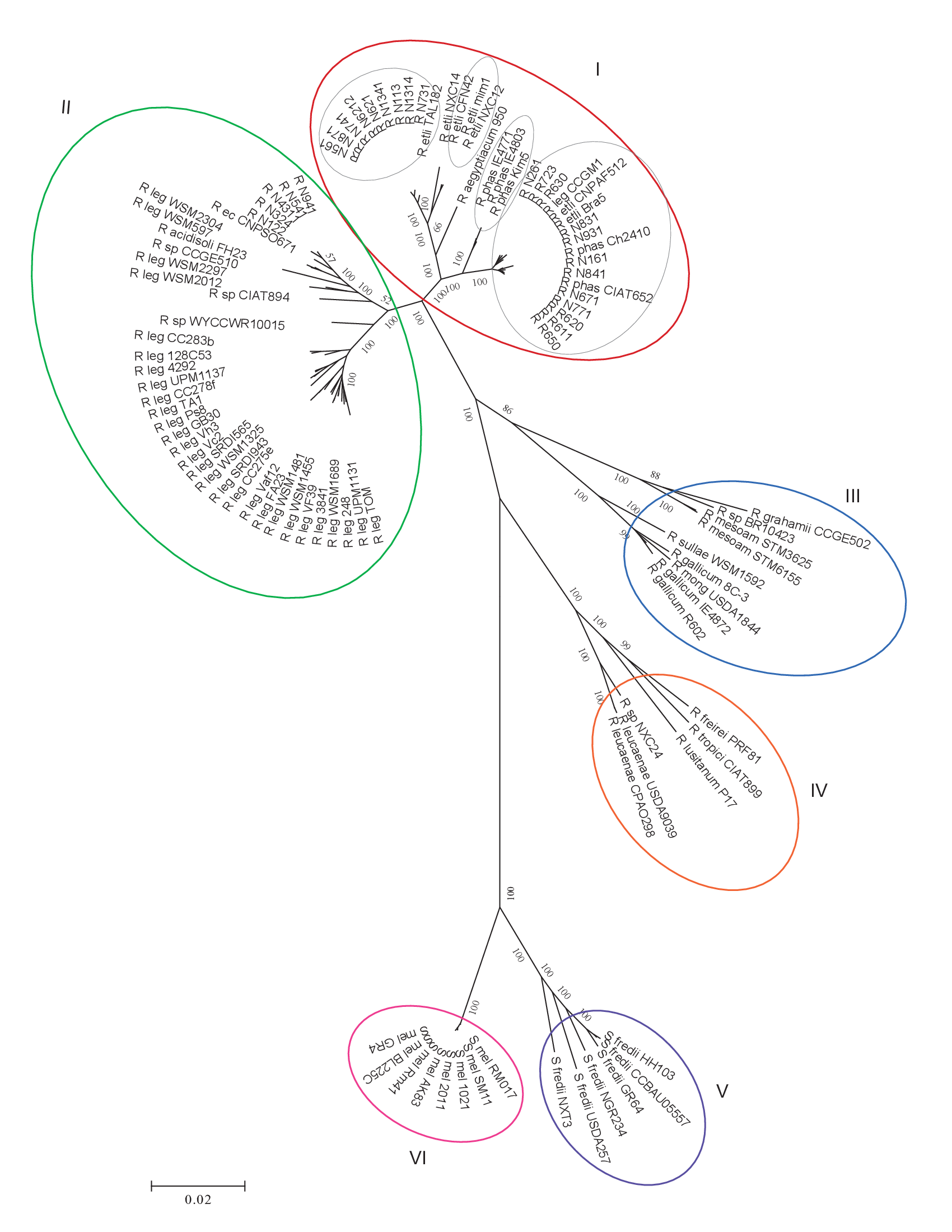

Supplement: FIGURE S1 — Phylogenetic tree of ribosomal proteins of Rhizobium species. Ribosomal clades are indicated by Roman numerals (I to VI) enclosed in colored ellipses. Subclades appear with black ellipses within the clades. The tree was constructed using maximum likelihood in the MEGA software package, by using 58 concatenated ribosomal proteins (refer to section “Materials and Methods” for more details). [file Image_1.TIFF]

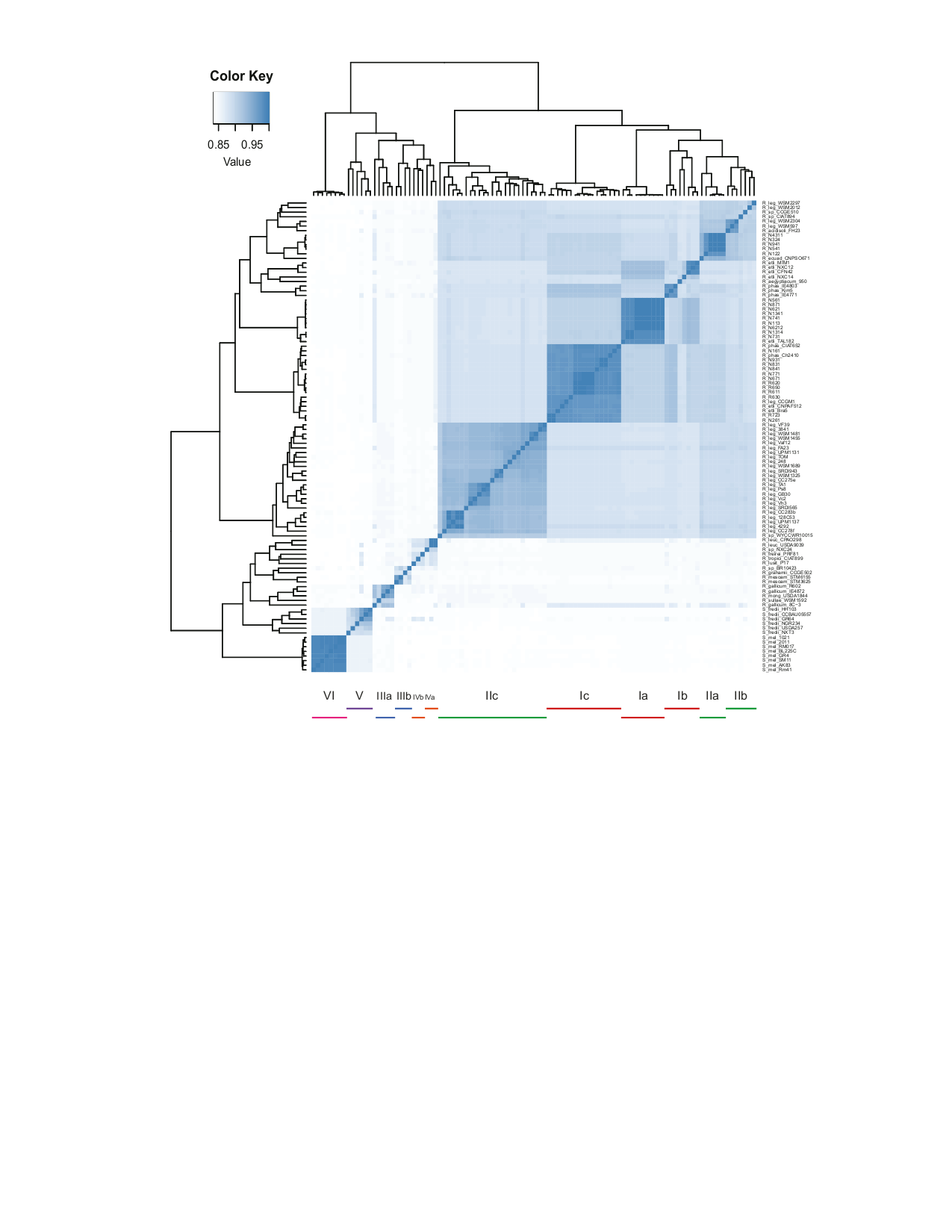

Supplement: FIGURE S2 — Genomic clusters of 102 Rhizobium and Sinorhizobium pairs obtained by pairwise ANIm comparisons. [file Image_2.TIFF]

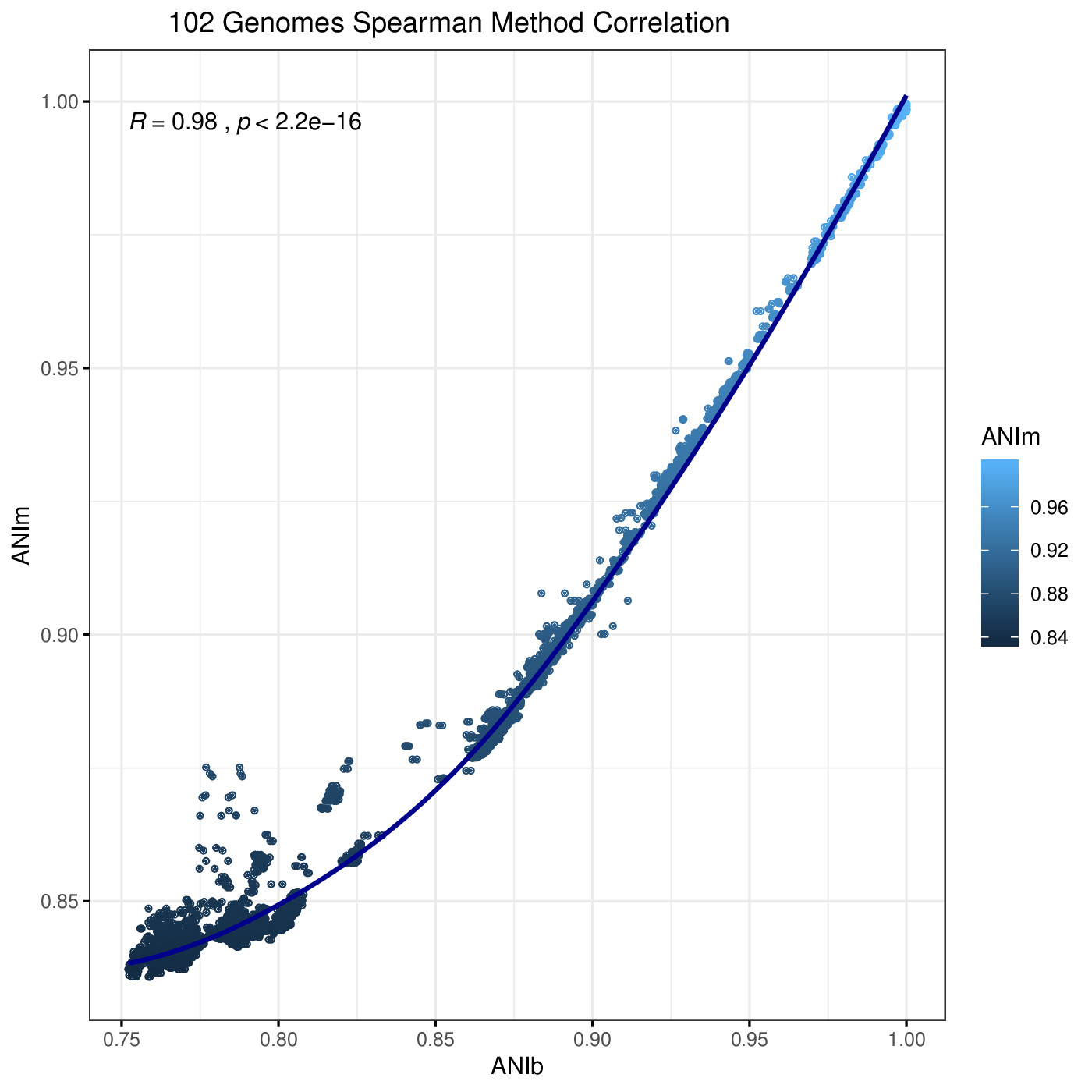

Supplement: FIGURE S3 — Correlation between ANIm and ANIb in pairwise comparison of 102 Rhizobium and Sinorhizobium genomes. Spearman r and p-values are shown. [file Image_3.TIFF]

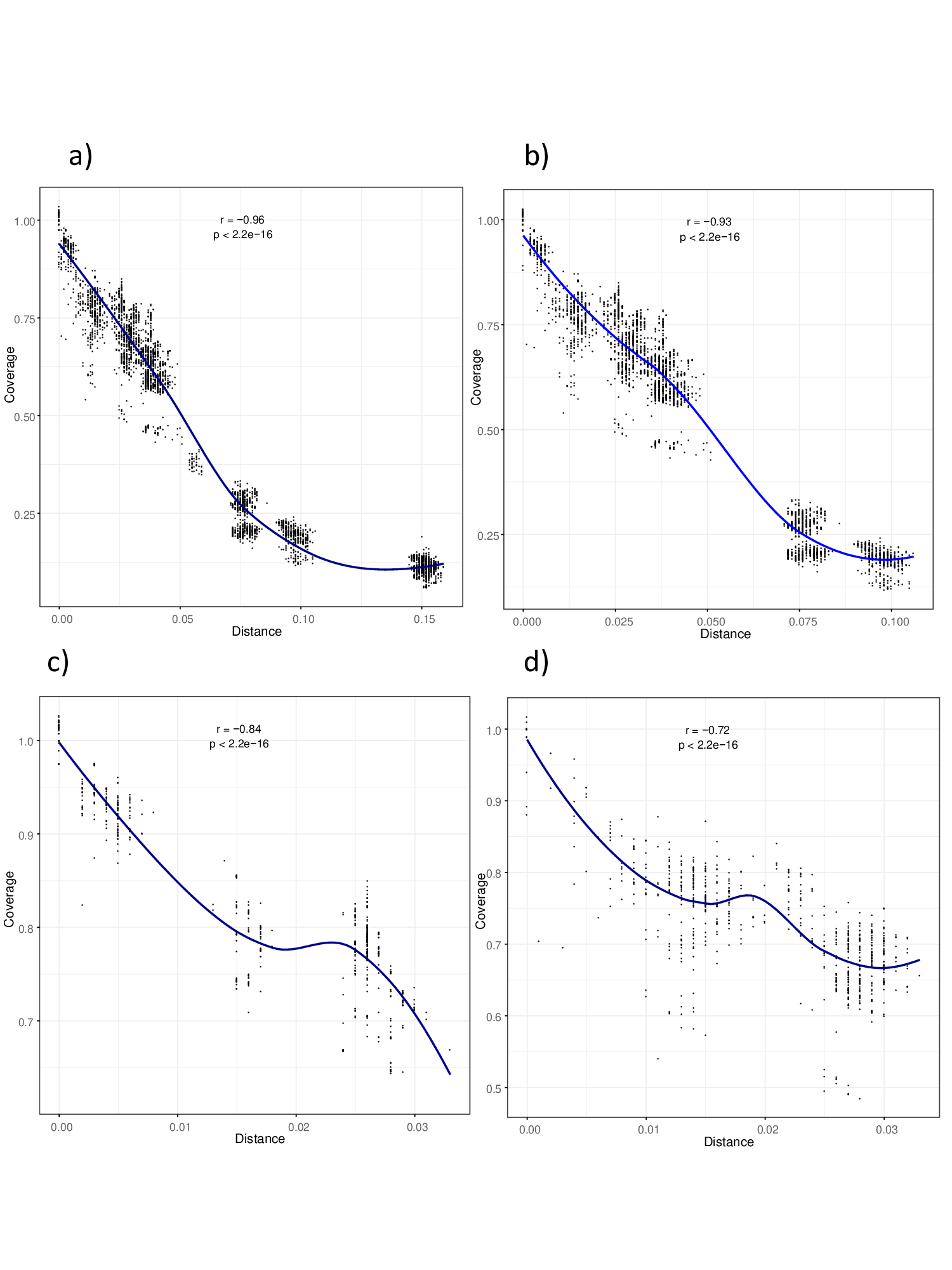

Supplement: FIGURE S4 — Correlation between Gcov and phylogenetic distance. (a) 102 Rhizobium and Sinorhizobium genomes, (b) 88 genomes, (c) 35 genomes of rC-I clade, and (d) 38 genomes o rC-II clade. Spearman r and p-values are shown. [file Image_4.TIFF]

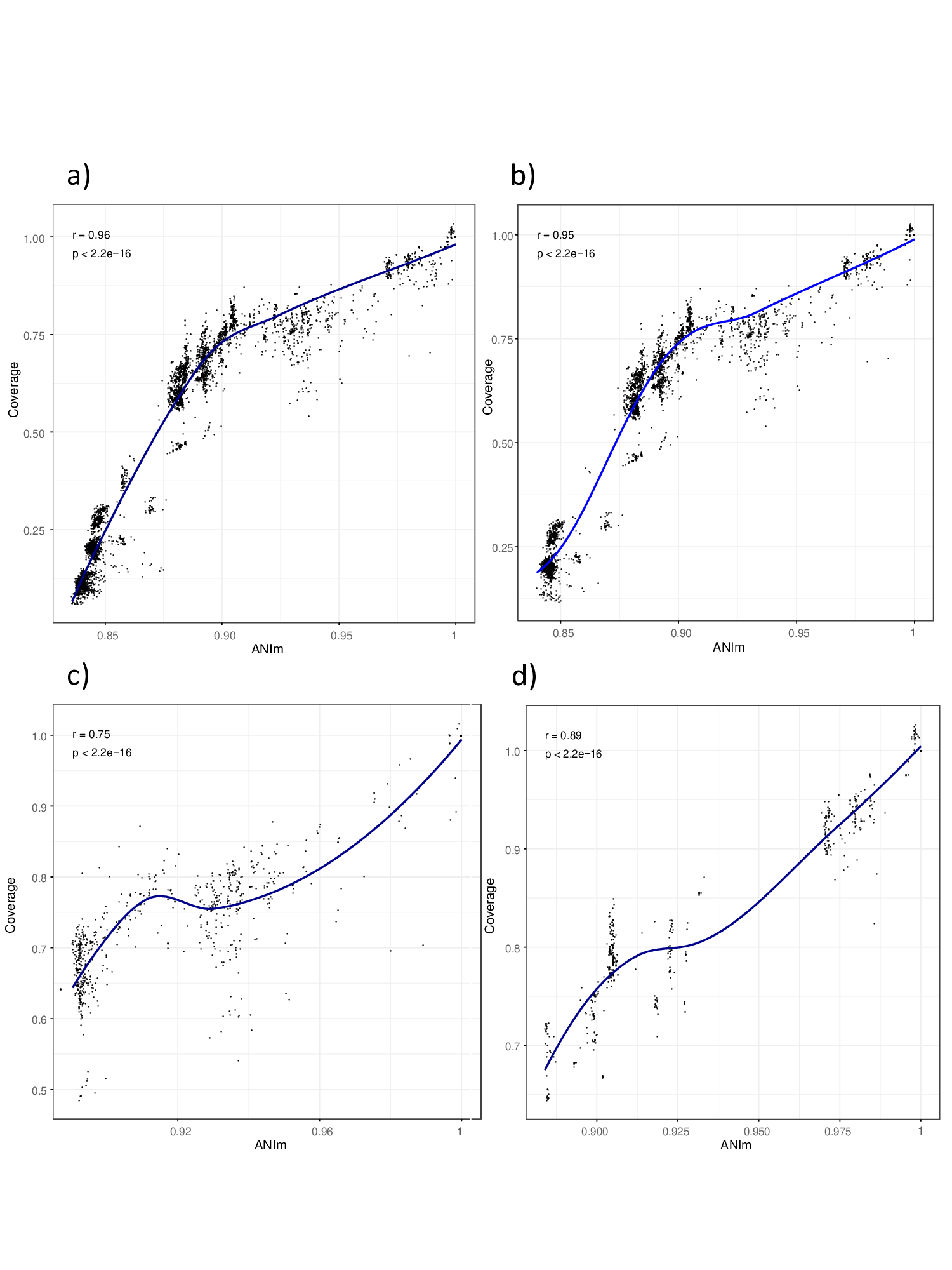

Supplement: FIGURE S5 — Correlation between ANIm and Gcov. (a) 102 Rhizobium and Sinorhizobium genomes, (b) 88 genomes, (c) 35 genomes of rC-I clade, and (d) 38 genomes o rC-II clade. Spearman r and p-values are indicated in the inset. [file Image_5.TIFF]

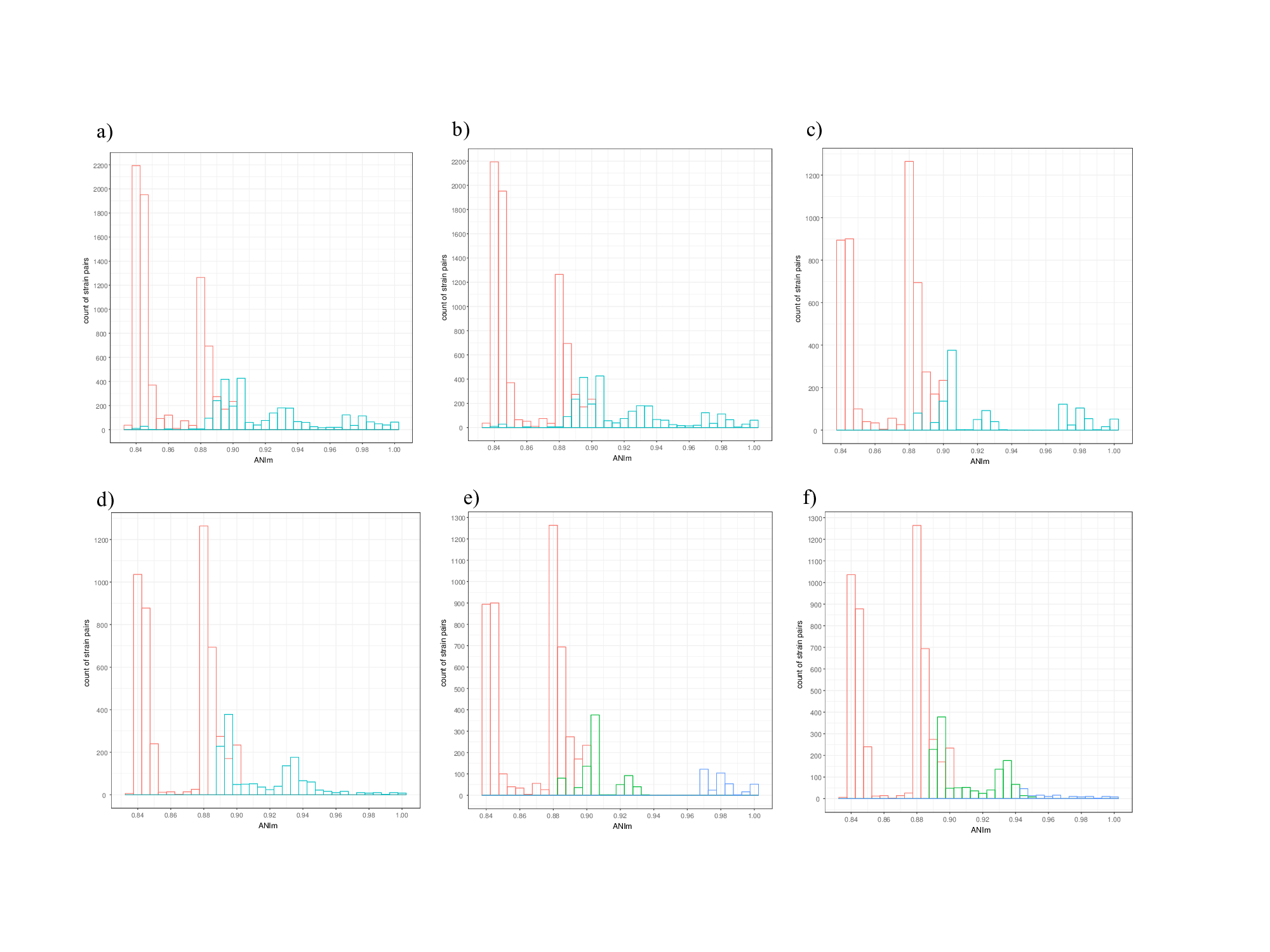

Supplement: FIGURE S6 — Distribution of ANI values between pairs of genomes belonging to (a) 102 genomes, (b) 88 genomes, (c) and (e) 35 genomes of rC-I clade, and (d) and (f) 38 genomes o rC-II clade. Histograms show the frequency of pairwise comparisons within clades (turquoise color), and inter clades (pale red), distributed by segments of ANI values. In figures (e) and (f), blue color histograms show comparisons within subclades (see Supplementary Figure S1) defined by ANIm > 95%, and the green color histograms indicate inter subclades comparisons. [file Image_6.TIFF]

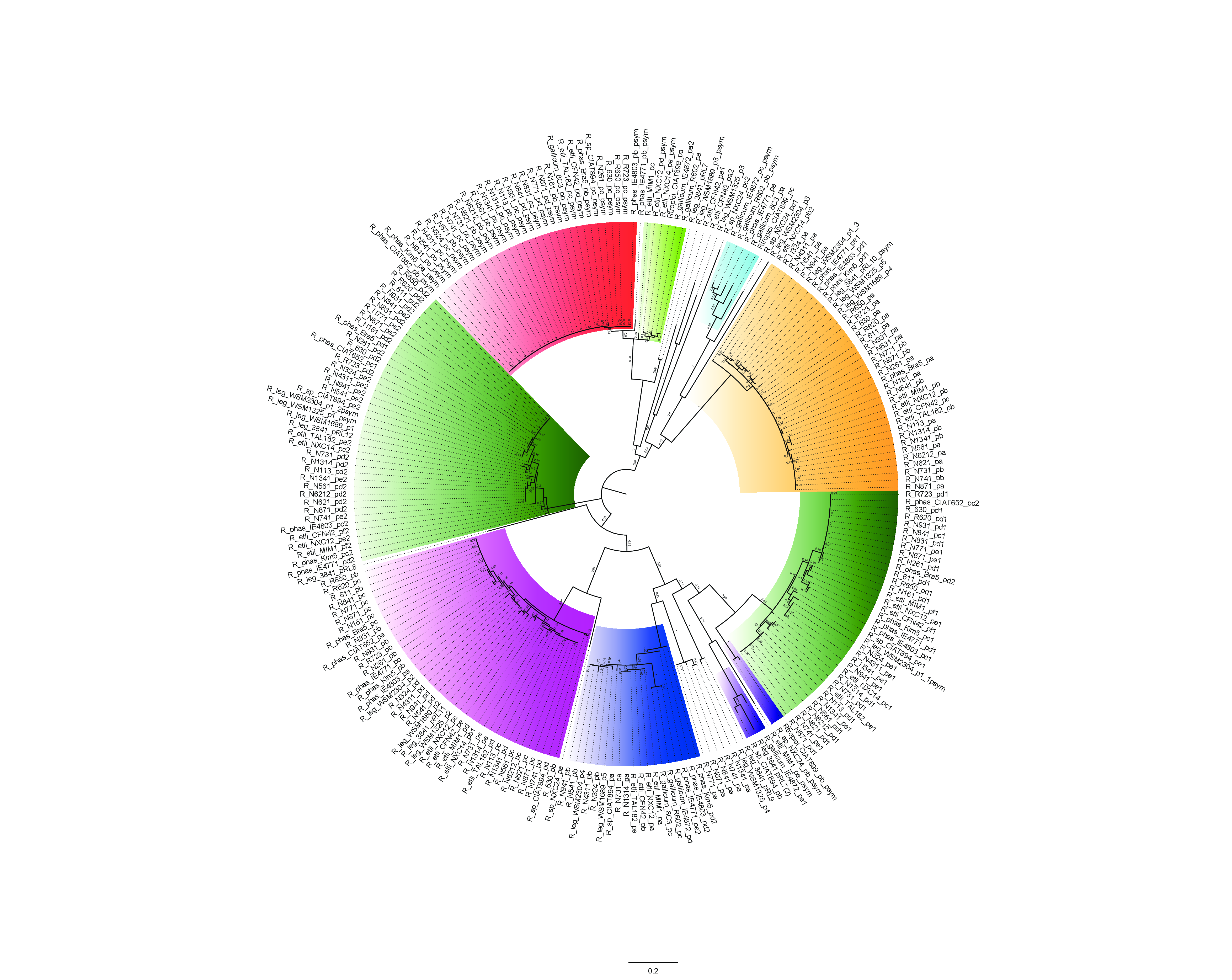

Supplement: FIGURE S7 — Phylogenetic families of RepB. Phylogenies were constructed with the maximum likelihood method and a bootstrap of n = 1000 replicates. Colors indicate the phylogenetic clades and their correspondence with the MCL clusters determined as indicated in the section “Materials and Methods” and Figure 5. [file Image_7.TIFF]

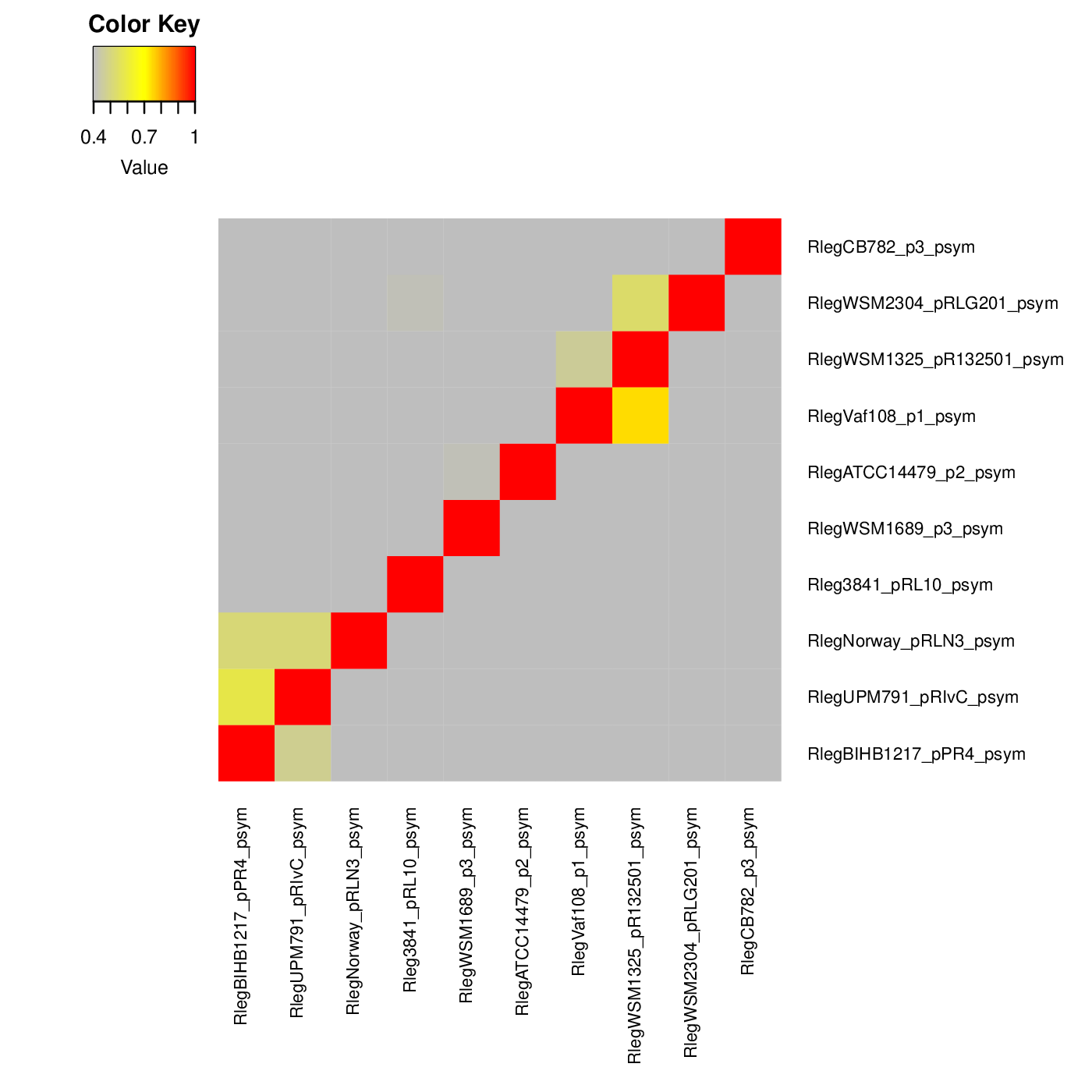

Supplement: FIGURE S8 — Pairwise ANIm comparison between complete R. leguminosarum symbiotic plasmids. Only the genome sequences of R. leguminosarum listed in the GenBank up to 14-03-2019 were downloaded. The ANIm comparison were performed using JSspecies as described in the methods section. The heatmap show the values of the corrected Gcov% / ANIm%; the color scale key inset shows the ranges from 0 to 1. [file Image_8.TIFF]

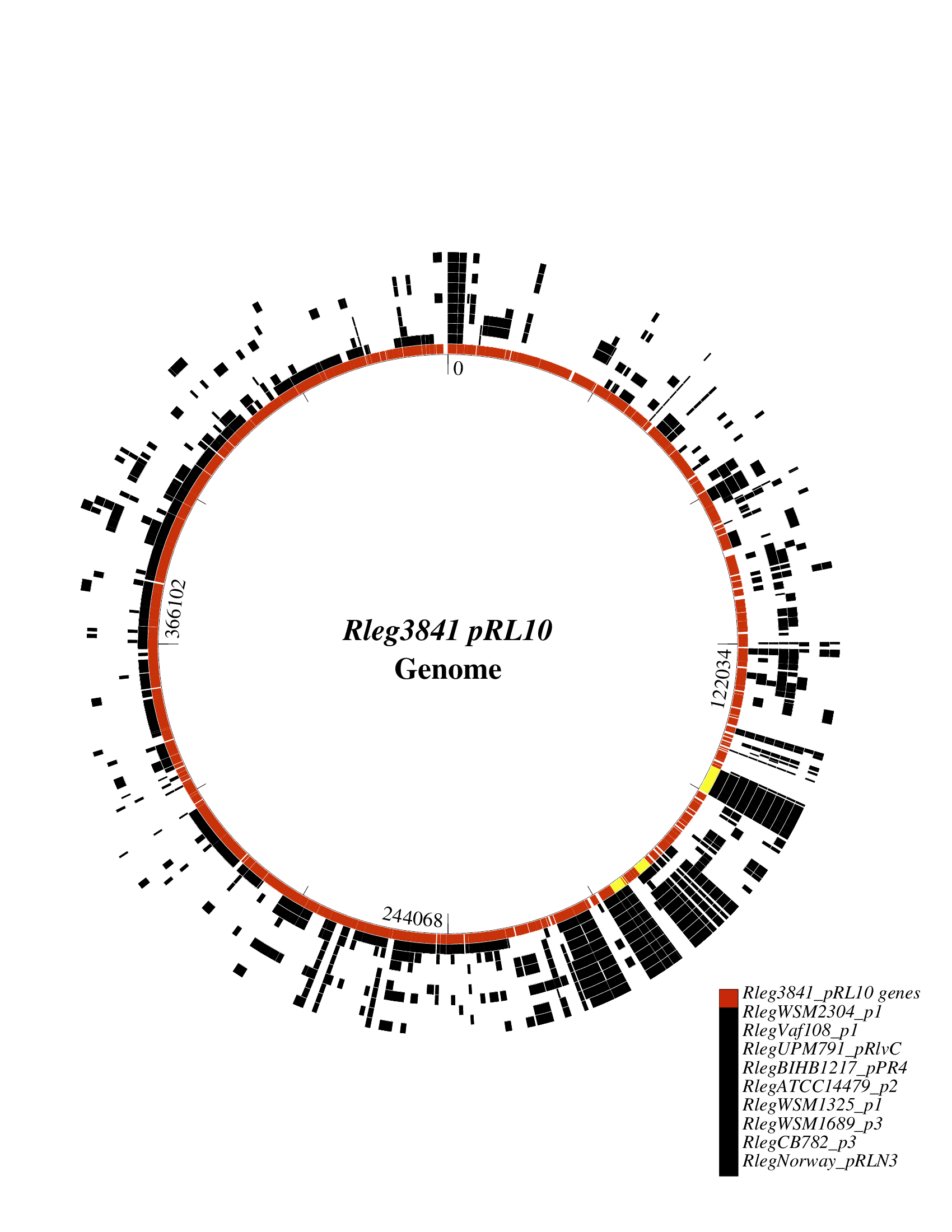

Supplement: FIGURE S9 — Comparison of the complete proteomes of the symbiotic plasmids of R. leguminosarum. Unidirectional BLASTp using the total predicted proteins of R. leguminosarum 3841 pRL10 symbiotic plasmid (illustrated by the red innermost circle) against the predicted proteins of others symbiotic plasmid indicated in the inset. Blast hits were recorded and showed in black circles. The nif-nod region is indicated by the yellow bars in the red inner circle. [file Image_9.TIFF]
